# The forgotten variable? Does the euthanasia method and sample storage condition influence an organisms transcriptome – a gene expression analysis on multiple tissues in pigs

**DOI:** 10.1186/s12864-023-09794-4

**Published:** 2023-12-14

**Authors:** B. Chakkingal Bhaskaran, R. Meyermans, W. Gorssen, G. E. Maes, J. Buyse, S. Janssens, N. Buys

**Affiliations:** 1 Department of Biosystems, Centre for Animal Breeding and Genetics, KU Leuven, Kasteelpark Arenberg 30, Box 2472, Leuven, 3001 Belgium; 2 Centre for Human Genetics, Genomics Core, UZ-KU Leuven, Leuven, Belgium; 3 Department of Biosystems, Laboratory of Livestock Physiology, KU Leuven, Kasteelpark Arenberg 30, Box 2472, Leuven, 3001 Belgium

**Keywords:** Nitrogen Anoxia, Pigs, T-61®, RNA*later*™, Liquid Nitrogen, Differential gene expression, Quantseq, Translational research

## Abstract

**Background:**

Transcriptomic studies often require collection of fresh tissues post euthanasia. The chosen euthanasia method might have the potential to induce variations in gene expressions that are unlinked with the experimental design. The present study compared the suitability of ‘nitrogen gas in foam’ (ANOXIA) in comparison to a non-barbiturate anaesthetic, T-61® (T61), for euthanizing piglets used in transcriptome research. Further, the effect of common tissue storage conditions, RNA*later*™ (RL) and snap freezing in liquid nitrogen (LN_2_), on gene expression profiles were also analysed.

**Results:**

On comparison of the 3’mRNA-Seq data generated from pituitary, hypothalamus, liver and lung tissues, no significant differential expression in the protein coding genes were detected between the euthanasia methods. This implies that the nitrogen anoxia method could be a suitable alternative for euthanasia of piglets used in transcriptomic research. However, small nuclear RNAs (snRNAs) that constitute the eukaryotic spliceosomal machinery were found to be significantly higher (log2fold change ≥ 2.0, and adjusted p value ≤ 0.1) in pituitary samples collected using ANOXIA. Non-protein coding genes like snRNAs that play an important role in pre-mRNA splicing can subsequently modify gene expression. Storage in RL was found to be superior in preserving RNA compared to LN_2_ storage, as evidenced by the significantly higher RIN values in representative samples. However, storage in RL as opposed to LN_2_, also influenced differential gene expression in multiple tissues, perhaps as a result of its inability to inhibit biological activity during storage. Hence such external sources of variations should be carefully considered before arriving at research conclusions.

**Conclusions:**

Source of biological variations like euthanasia method and storage condition can confound research findings. Even if we are unable to prevent the effect of these external factors, it will be useful to identify the impact of these variables on the parameter under observation and thereby prevent misinterpretation of our results.

**Supplementary Information:**

The online version contains supplementary material available at 10.1186/s12864-023-09794-4.

## Background

Transcriptome studies in pigs have increased over the past decade, aiming to interpret tissue specific processes that control significant traits related to growth, immunity and disease development [1–4]. Due to the closeness to human physiology and anatomy as well as the genomic similarities, pigs have been considered as a suitable model for translational research [5–8]. Several studies have been using pigs as animal models for preclinical studies in humans [9, 10].

Transcriptome studies often require collection of tissues post euthanasia. The recommendations by the European Council [11, 12] as well as the guidelines from the American Veterinary Medical Association [13] enlist several criteria on the choice of euthanasia methods in experimental animals. For the selection of euthanasia method, prime consideration must be given to ensure the welfare of the animals. Additionally, the chosen method should not influence subsequent evaluation of the parameter under investigation. The euthanasia method should keep the tissue of interest intact and more importantly, it should not cause a source of variation in the gene expression profile. Alike euthanasia methods, tissue storage condition has also got the potential to induce variations in gene expressions, that are unlinked with the experimental design [14–17]. This can further introduce a bias in the top gene expression rankings and subsequently mask the experimental effects. Hence, in the context of transcriptomic research, it is important that the (m)RNA levels related to the tissue under investigation remain unlinked to the method of euthanasia and tissue storage conditions used in the experiment.

Recently, we examined the effect of an inhalant anaesthetic ‘nitrogen gas in foam’ (ANOXIA) on RNA yield and quality parameter in piglets and compared it against an injectable anaesthetic T-61® (T61)[18]. We combined it with two different storage conditions—RNA*later*™ (RL) vs snap freezing in liquid nitrogen (LN_2_). It was found that ANOXIA could be used as a suitable alternative to T61 method based on RNA quality parameters, while storage in RL significantly increased RNA integrity when compared to LN_2_ storage method. Other studies investigated the effect of various euthanasia methods on protein kinases [19] and brain mRNA levels in mice [20, 21]. In comparison to carbon dioxide (CO_2_) asphyxiation, euthanasia by decapitation without anaesthesia or with ketamine/xylazine or isoflurane anaesthesia induced significant phosphorylation of mitogen activated protein kinases, highlighting a ‘euthanasia-instigated’ difference in the protein kinases activity. Similarly, ‘focused beam microwave irradiation’ method of euthanasia in mice significantly reduced brain mRNA expression levels, compared to euthanasia by CO_2_ inhalation. Naïve mice and mice with acute traumatic ‘induced’ brain injury showed varying levels of gene expression in hippocampus when evaluating the effect of euthanasia methods like isoflurane inhalation, chloralhydrate injection and a combination of anaesthetics against an effect of no anaesthesia. Also, the impact of anaesthesia and euthanasia on metabolomics of different mammalian tissues, using a mouse model, has been previously documented [22]. Most notably, elevated levels of multiple nucleotide and purine degradation metabolites were present in skeletal muscle, heart and liver tissues of euthanized animals, indicating deteriorating effect of euthanasia on nucleotides, on comparison with tissues from anaesthetized animals. In pigs, the effect of physical and inhaled euthanasia methods on hormonal measures of stress was previously examined [23], where gradual administration of CO_2_ or 70% N_2_/30% CO_2_ produced similar plasma concentrations of stress indicators to that of physical euthanasia methods.

To our knowledge, no previous research was done at transcriptome level to measure gene expression profiles in porcine tissues that compares the effect of different euthanasia methods and storage conditions. Tissues meant for transcriptome studies are often snap frozen in LN_2_ after collection and eventually stored at -80 °C until RNA extraction. Due to several advantages over the snap freezing method like improved RNA yield, quality and integrity as well as ease of use in field conditions [18, 24–26], a commercial preparation of ammonium salt solution, RNA*later*™ (RL), is now popularly used for preserving tissues for gene expression studies. However, there are also recent studies that reported the influence of RL on gene expression. Comparison of microarray profiles from adenocarcinoma tissues in humans indicated that around 2.3% (corresponding to 540 genes) of the total expressed genes displayed more than a two-fold difference in the RL stored samples compared to the snap frozen samples. The upregulated genes were those involved in translational regulation and RNA metabolism and the ones that were downregulated were associated with regulation of enzymatic and energy-dependent processes [14]. Another study involving albino rats reported a significant influence of RL storage on gene expression levels in multiple tissues, measured by qPCR analysis[15]. It was reported that despite being able to maintain RNA integrity, RL is unable to inhibit biological activity and thereby provide a representative gene expression, compared to storage in LN_2_. A similar finding was also described in a RNAseq study in 30 day old fries (young fish with unabsorbed yolk sac) suggesting a potential role of RL in eliciting a response that can bias the gene expression[16]. Further, evidences for differential expression and post translational modifications were observed in RL’ stored *Arabidopsis thaliana* tissues, using RNAseq[17]. Hence, it is important to investigate if the storage condition and its interaction with the method of euthanasia would introduce a variation that is not connected to the experimental condition under investigation.

Quantseq is a robust mRNA sequencing method used in transcriptomics. The quantseq 3’ mRNA-Seq (also known as 3’ RNA Tag-Seq method) targets the 3’ poly-A tail of mRNA [27–29]. It is cost effective as well as devoid of fragment size bias because it generates a uniform read distribution irrespective of the original RNA length. Compared to whole transcriptome sequencing, quantseq is a suitable alternative especially to quantify gene expression and measure the differential gene expression between two biological conditions, at relatively low sequencing depth (~ 2 million reads per sample) [24–26].

The aim of the current study is to investigate whether the choice of euthanasia method (ANOXIA method vs T61), together with the effect of the sample storage condition (RL vs LN_2_) had an influence on the gene expression profile in pigs. For this, we compared the RNAseq data (quantseq 3’ mRNA) obtained from brain, lung and liver tissues and checked whether the gene expression profiles were affected by the experimental conditions. These tissues were considered the most relevant ones for this study because brain and lung will be exposed immediately to these euthanizing agents eliciting a response whereas liver will play a pivotal role in its metabolism. With this research, we want to highlight the importance of these forgotten variables that can introduce biological or non-biological variations in transcriptomic studies.

## Results

### Descriptive statistics – RNA extraction

All tissues used in this experiment were sampled and stored within a mean sampling time of 7.75 min (Standard Deviation (SD) = 1.44, Interquartile Range (IQR) 6.40–9.10), although there were differences in sampling time between tissue types (Table 1). High quality RNA was extracted from these tissues and had an average concentration of 674.7 ng/ µl (SD 379.6, IQR 361.4–847.5) RNA. RNA quality parameters comprising A260/230 ratio and A260/280 ratio were 1.99 (SD 0.29, IQR 1.93–2.18) and 2.11 (SD = 0.02, IQR 2.08- 2.13) respectively. The integrity of RNA measured as RIN value had an average measurement of 8.81 (SD = 1.15, IQR 8.28–9.70). A detailed description of the experimental data, based on grouping by euthanasia method, storage condition and tissue types is given in Table 1.

**Table 1:** RNA measurements and sequencing summary on tissues grouped by euthanasia method and storage condition

**Characteristic**	**N**	**Overall**	**Anoxia**	**T61**	**LN**_**2**_	**RNA*later***	**Hypothalamus**	**Liver**	**Lungs**	**Pituitary**
		*N* = 59^1^	*N* = 30^1^	*N* = 29^1^	*N* = 23^1^	*N* = 36^1^	*N* = 16^1^	*N* = 16^1^	*N* = 15^1^	*N* = 12^1^
**Storage Condition**	59									
LN_2_		23	12	11			8	8	7	0
RNA*later*		36	18	18			8	8	8	12
**Euthanasia Method**	59									
Anoxia		30			12	18	8	8	8	6
T61		29			11	18	8	8	7	6
**Tissue Type**	59									
Hypothalamus		16	8	8	8	8				
Liver		16	8	8	8	8				
Lungs		15	8	7	7	8				
Pituitary		12	6	6	0	12				
**Time of sampling (minutes)**	59	7.75 (1.44)	7.98 (1.18)	7.51 (1.65)	7.80 (1.50)	7.71 (1.41)	6.67 (1.02)	8.67 (1.39)	8.00 (1.45)	7.63 (1.06)
**RNA Concentration (ng/µl)**	59	674.7 (379.6)	732.1 (390.9)	615.4 (364.9)	607.4 (372.6)	717.7 (383.0)	444.7 (263.4)	838.4 (437.8)	568.1 (289.1)	896.4 (335.6)
**A260_230 ratio**	59	1.99 (0.29)	2.02 (0.22)	1.96 (0.34)	1.98 (0.21)	2.00 (0.33)	2.02 (0.21)	1.91 (0.28)	1.92 (0.40)	2.16 (0.12)
**A260_280 ratio**	59	2.11 (0.02)	2.11 (0.03)	2.10 (0.02)	2.12 (0.02)	2.10 (0.02)	2.11 (0.03)	2.10 (0.02)	2.12 (0.02)	2.09 (0.02)
**RIN**^a^	56	8.81 (1.15)	8.85 (1.00)	8.77 (1.32)	7.99 (1.40)	9.26 (0.66)	7.56 (1.22)	9.43 (0.61)	9.31 (0.86)	9.18 (0.33)
**Reads (million)**	59	2.89 (0.38)	2.82 (0.37)	2.97 (0.37)	2.90 (0.33)	2.89 (0.41)	2.99 (0.37)	2.79 (0.41)	2.85 (0.34)	2.96 (0.38)
**Alignment (%)**	59	84.86 (1.27)	84.81 (1.30)	84.92 (1.26)	84.56 (1.33)	85.06 (1.21)	84.05 (0.90)	85.93 (1.24)	84.29 (1.0)	85.25 (0.91)
**Unique alignment (%)**	59	71.5 (3.8)	71.2 (3.7)	71.7 (3.9)	69.9 (3.3)	72.5 (3.8)	67.5 (2.1)	71.4 (4.2)	74.1 (2.2)	73.5 (1.6)
**Aligned multimapping (%)**	59	13.4 (3.4)	13.6 (3.1)	13.2 (3.8)	14.7 (2.9)	12.6 (3.5)	16.5 (2.2)	14.6 (3.3)	10.2 (2.1)	11.8 (1.2)
**Non-aligned (%)**	59	15.14 (1.27)	15.19 (1.30)	15.08 (1.26)	15.44 (1.33)	14.94 (1.21)	15.95 (0.90)	14.07 (1.24)	15.71 (1.00)	14.75 (0.91)

^1^n; mean and the respective standard deviation is given in brackets. ^a^RIN measurement was undetermined for three samples, despite good RNA quality parameters. However, good quality reads were obtained following sequencing. RNA yield couldn’t be calculated as tissue weights were not known for all the tissues, although the samples were of an approximate dimensions of a 3mm^3^ cube, weighing less than 30 mg

### Summary of sequencing data

Raw sequence data from pituitary, hypothalamus, lung and liver were processed for further analysis. After sequencing, adapter and low quality sequences were trimmed off to attain clean reads. On average, 2.89 ± 0.38 million reads per sample were retained after quality control, and used for further analysis. The reads after alignment to the Sscrofa11.1 genome had an overall alignment rate of around 84.9 ± 1.27%, which included 71.5 ± 3.8% uniquely mapped reads and 13.5 ± 1.27% of the reads mapping to multiple locations on the genome. A summary of sequencing on the basis of grouping by experimental conditions and tissue type is described in Table 1. All the parameters described here had comparable values between the euthanasia methods. However, analysis of variance (ANOVA) revealed a significant effect (p < 0.001) of tissue types and storage conditions on the ‘unique alignment’ percentage (Supplementary Table S1, Additional File 1). Post hoc analysis indicated that the hypothalamus samples (67.5 ± 2.1%) (Fig S1) and samples stored in LN_2_ (69.9 ± 3.3%) (Fig S2) had a significantly lower ‘unique alignment’ percentage. A detailed description of the sequencing data from all the samples used in this study is given in the supplementary Table S2, Additional File 2.

### Differential gene expression analysis

**Fig. 1:**
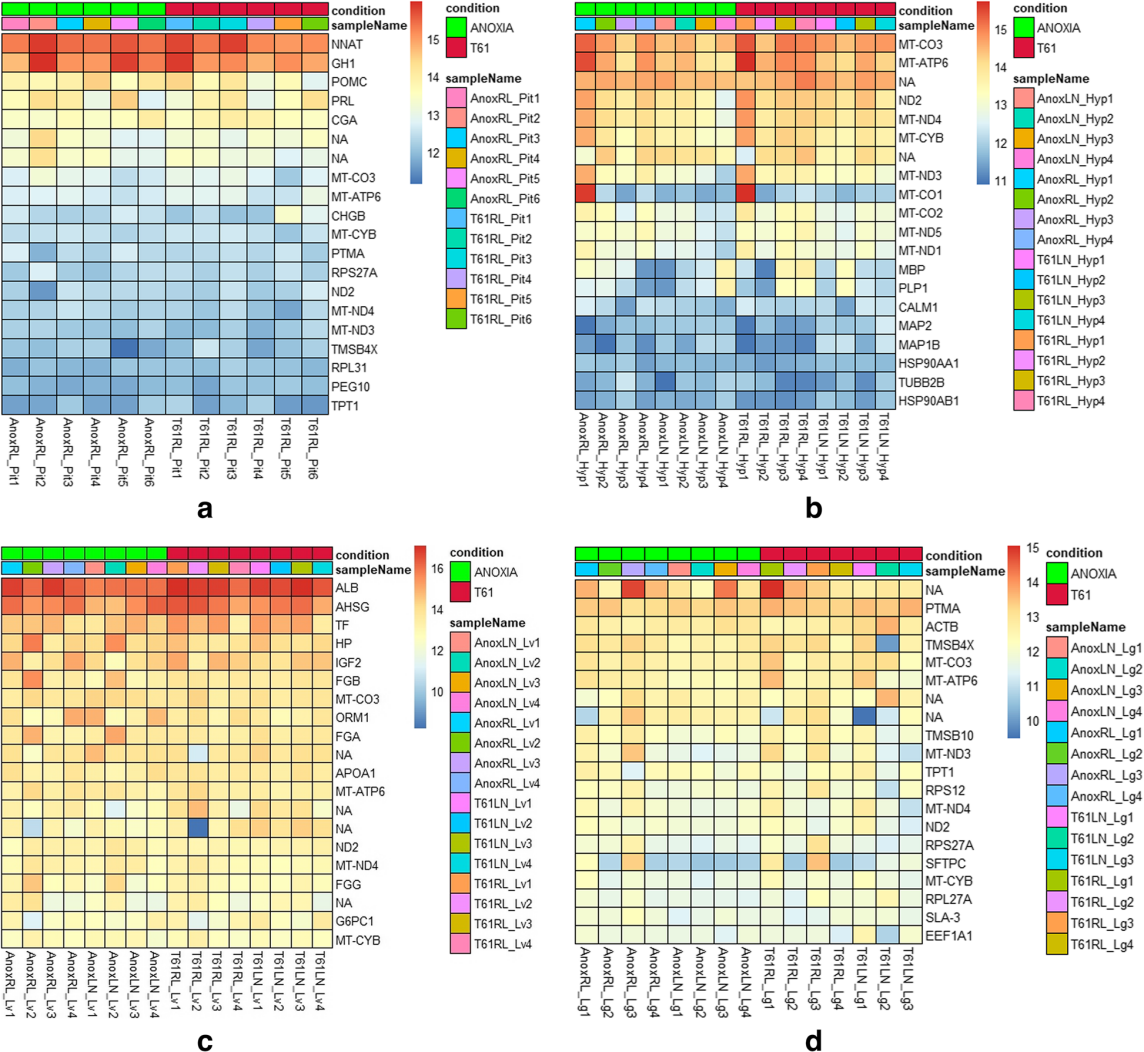
Heat maps of gene expression data from pituitary, hypothalamus, liver and lungs samples (Fig A- D) comparing the two euthanasia methods, ANOXIA and T61. Each row represents a gene and each column represents a sample. The top 20 genes ranked based on magnitude of gene expression are depicted and the colours of the tiles represent the measured expression value of a gene in that sample. The data is normalized using the variance stabilization transformation. The samples are named by combining the euthanasia method and storage condition, followed by the tissue type of the sample For example, AnoxRL_Lv is a liver sample, from an animal euthanized by ANOXIA and stored in RNA*lat*er. The numbers marked on the sample name indicates x^th^ animal per condition

Count data generated following sequence analysis were used for estimating differential gene expression in different tissue types. Pituitary, hypothalamus, lung and liver samples, grouped based on euthanasia method and storage condition, were evaluated for identifying differentially expressed genes. Expression levels of the top 20 highly expressed genes across samples specific to different tissue types is represented in the heatmap (Fig. 1 A –  D). The samples (AnoxRL_Hyp1 and T61RL_Hyp1) representing hypothalamus tissues from two animals, each euthanized by ANOXIA and T61 respectively and stored in RL (Fig. 1 B), showed a different level of gene expression especially for the mitochondrial gene mt*CO1*. This gene encodes for the cytochrome C oxidase subunit 1, predicted to be involved in mitochondrial electron transport. Barring this deviation, the overall gene expression profile suggests that there is no influence of the euthanasia method on the expression of top ranked genes across different tissues types.

### Effect of euthanasia methods and storage condition on gene expression profile in pituitary and hypothalamus

**Fig. 2:**
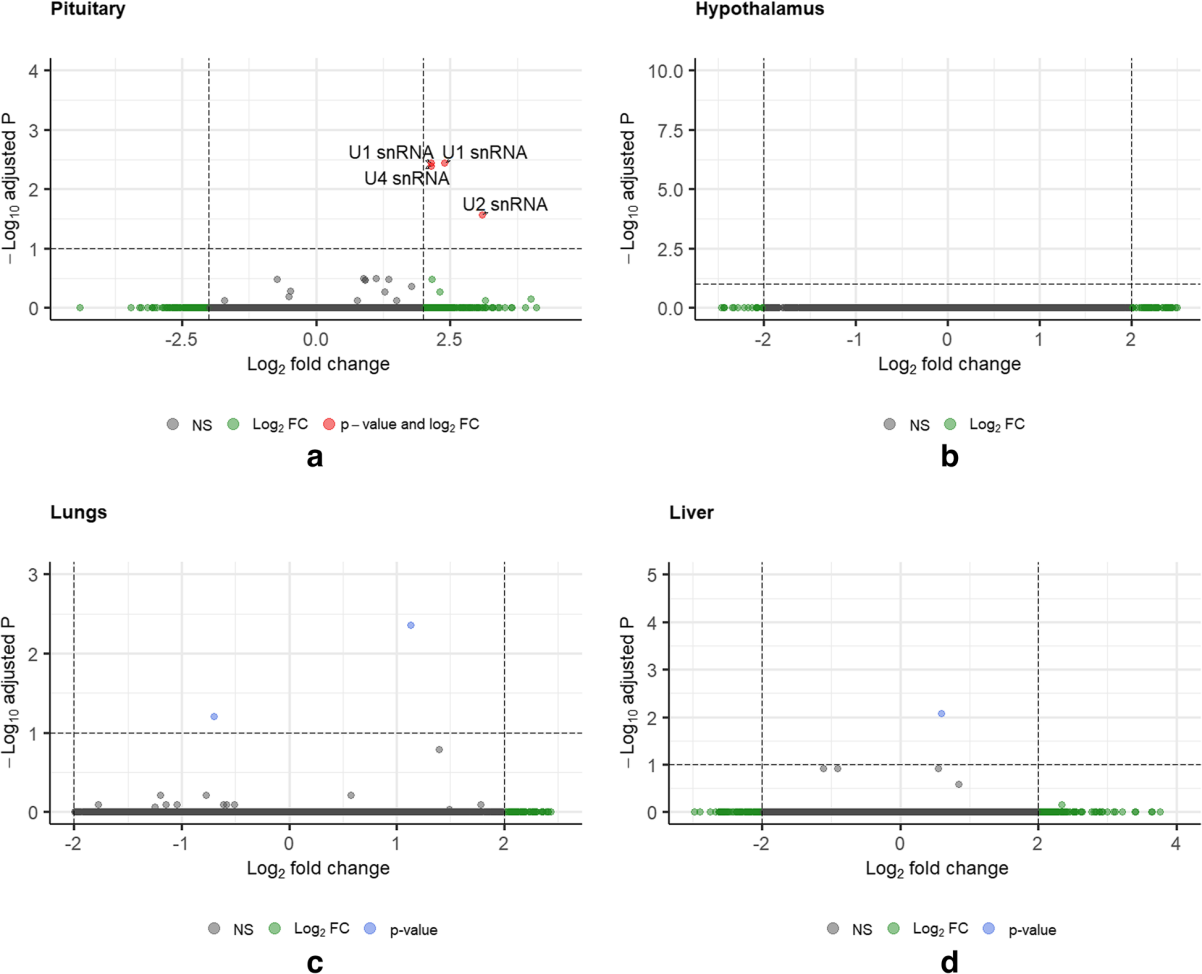
Enhanced volcano plot identifying differentially expressed genes between the euthanasia methods in different tissue types. In pituitary, no significant deviation in expression of protein coding genes was detected. However, small nuclear RNAs (snRNAs) were found to be significantly higher while using the ANOXIA method (Fig. 2A). No significant differential gene expression was observed in hypothalamus (Fig. 2B), lungs (Fig. 2C) and liver (Fig. 2D). Log2 fold change is plotted on the x axis and negative logarithm (to the base 10) of adjusted p-value is plotted on the y axis

Samples from pituitary were stored exclusively in RL and hence the gene expression analysis from this tissue is a direct comparison of the two euthanasia methods, ANOXIA and T61. Differential Gene Expression (DGE) analysis on the gene count data from the pituitary did not identify any statistically significant expression differences between the two euthanasia methods. Out of the 16,777 ‘non zero’ genes from the count matrix that were mapped to the pig genome, none of the protein coding genes were differentially expressed. Although there were no protein coding genes differentially expressed across the two conditions, small nuclear RNAs (snRNAs) namely U1, U2 and U4 that constitute the eukaryotic spliceosomal machinery were found to be significantly higher (Log2 Fold Change (LFC) ≥|2.0|, and adjusted p value ≤ 0.1) in pituitary samples belonging to the ANOXIA group in comparison to T61 (Fig. 2 A).

**Fig. 3:**
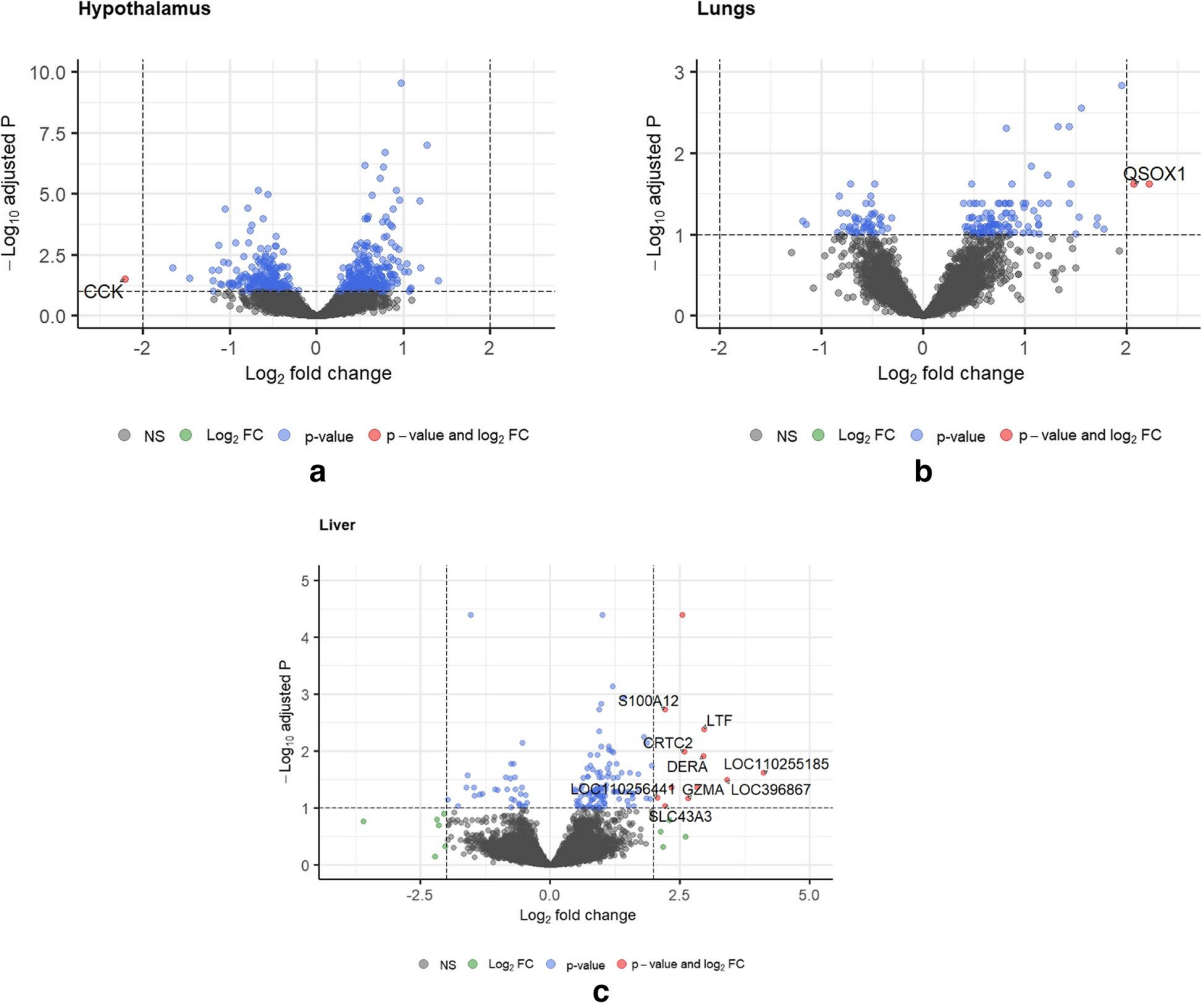
Enhanced volcano plot identifying differentially expressed genes between the storage conditions, from the tissue types Hypothalamus(Fig. 3A), Lungs (Fig. 3B) and Liver (Fig. 3C). Log_2_ fold change is plotted on the x axis and negative logarithm (to the base 10) of the adjusted p value is plotted on the y axis. Several genes were differentially expressed in liver samples stored in RNA*later*

In the hypothalamus, no significant effect of the euthanasia methods on the gene expression profile was observed (Fig. 2 B). Out of the 16,599 ‘non zero’ genes that mapped to the pig genome, there was no differential expression detected. However, while comparing the storage methods, it was noticed that the transcripts of the gene cholecystokinin (*CCK*) appeared to be significantly lower (LFC ≥|2.0| and adjusted p value ≤ 0.1) in the hypothalamus samples stored in RL (Fig. 3A, (See supplementary Table S3, Additional File 3 for full list of genes). It is also noteworthy that no interaction effect of the euthanasia method and storage condition was detected on the gene expression profile in the hypothalamus.

### Effect of euthanasia methods and storage condition on gene expression profile in lungs and liver

The analysis of gene expression data from lungs mapped a total of 16,805 non zero count genes to the *Sus scrofa* genome. No genes were found to be differentially expressed at a threshold of LFC ≥|2.0| and adjusted p value ≤ 0.1 (Fig. 2C). Interestingly, the storage condition was found to be a significant factor that influences the detection of differentially expressed genes in lungs. Expression of two genes (quiescin sulfhydryl oxidase 1—*QSOX1* and ENSSSCG00000037150 (currently in ensemble archive) were found to be significantly higher (LFC ≥|2.0| and adjusted p value ≤ 0.1) in lung tissues stored in RL, as compared to LN2 (Fig. 3B).

For liver samples, no significant effect of the euthanasia method on gene expression was detected (LFC ≥|2.0| and adjusted p value ≤ 0.1) (Fig. 2D). Interestingly, method of tissue storage had a significant effect on gene expression in the liver tissue, similar to that in lungs. It was observed that out of the 15,778 ‘non zero’ genes from the count matrix mapped to the genome, twelve genes appeared to be upregulated (LFC ≥|2.0| and adjusted p value ≤ 0.1) in the liver tissues stored in RL in comparison with tissues that were snap frozen and stored in LN_2_ (Fig. 3C, See supplementary Table S4, Additional File 4 for the list of differentially expressed genes). Further, we did not detect any interaction effect of the euthanasia method and storage conditions on the gene expression profile in liver tissue. No genes were found to be differentially expressed when the interaction term was included in the model.

## Discussion

In transcriptome studies, different euthanasia methods and tissue storage conditions are routinely used and although this could affect gene expression levels that are unrelated to the experimental design, to our knowledge this has not been investigated so far in pigs. Moreover, the suitability of a method using nitrogen anoxia opposed to T61 injection for euthanizing piglets used in transcriptome research, was not previously tested. Due to the increasing use of pigs in translational research, we have to rule out whether this novel experimental condition can induce a biological variation due to the interaction with the individual or the storage condition.

The present study evaluated the effect of two euthanasia methods (ANOXIA and T61) and two storage conditions (RL and LN_2_) on the differential gene expression in pituitary, hypothalamus, liver and lungs of male piglets. The statistical model in the differential gene analysis also tested whether there was an interaction effect of these variables on the gene expression profile.

No significant differential expression of any of the known protein coding genes were observed in pituitary and hypothalamus samples, on comparison of the euthanasia methods. Also, for the lung and liver samples, there were only a couple of genes differentially expressed, notably at a very low log2fold change (LFC ≥|0.5|). Considering our small sample size per condition (*n* = 4), we would ideally set a higher LFC threshold as recommended by Schurch et al.[30], to be absolutely sure about the credibility of the differentially expressed genes. When set at a higher LFC threshold (LFC ≥|2.0|), the expression of these genes in hypothalamus and pituitary was found not to be significantly different between the euthanasia methods studied. When there was no or very little evidence against the null hypothesis, the false discovery rate was very close to one for most of the values and this was evident when the results were plotted with adjusted p value (Fig. 2 A-D). These above observations imply that the nitrogen anoxia method might be a suitable alternative for the T61 injection for euthanizing piglets used in transcriptomics research, as there weren’t any differential expression of protein coding genes. However, there was an interesting observation while analysing the transcriptomic data from the pituitary samples. Small nuclear RNAs (snRNAs) such as U1, U2 and U4, that form a key component of the spliceosomal machinery in the eukaryotes [31, 32], were found to be differentially expressed in pituitary, between the two euthanasia methods. These snRNAs were found to be significantly higher in pituitary samples belonging to the ANOXIA group in comparison to the T61 group.

Differential expression of snRNAs observed across the two conditions tested cannot be overlooked, given the influence of this category of non-coding RNAs on varied cellular processes [33]. These small RNA molecules are found in the cell nucleus and are involved in the processing of pre-messenger RNAs. The sm-class of snRNA genes (classified based on the binding site it possesses for small (Sm) proteins of less than 20 kDa in size) include U1, U2 and U4 snRNAs that play a significant role in catalysing the removal of introns from pre-mRNA [34, 35]. They perform this function by forming a complex with snRNA specific partner proteins that are classified as the small nuclear ribonucleoproteins (snRNP) [35].The gene ontology (GO) terms associated with U1 snRNA is mRNA 5'-splice site recognition and pre-mRNA splice site binding. Similarly, GO terms associated with U2 snRNA is mRNA branch site recognition and pre-mRNA branch point binding. U4 snRNA is also an important member of the spliceosomal process as it is involved in spliceosomal tri-snRNP complex assembly formation. It is also associated with formation of quadruple SL/U4/U5/U6 snRNP as well as U6 snRNA binding [36].

An increase in the level of snRNAs as observed in the nitrogen anoxia method indicates an altered cellular process where snRNA activity was increased. It could be interpreted that these genes were less stable or got degraded during euthanasia with T61 injection, probably as a response to the drug. As a consequence of the euthanasia method used, the splice variant of a gene is either formed or masked, depending on the up/down regulation of the snRNA. Either ways, the consequence of this change must be further investigated as it has the potential to modify the gene expression, like the effect of a splice variant, that we originally intent to measure. In humans, there are evidences for regulation of gene expressions by the U1 snRNAs at the pre-mRNA processing stage [37]. Multiple evidences for down regulation of numerous snRNA genes in response to UV light treatment has also been previously documented [31]. It is worth mentioning that only a small proportion of genes were differentially expressed due to the euthanasia method. However, given the extensive use of pigs in translational research, any effect in response to the genomic stress caused by the method of euthanasia must be ruled out before concluding the research findings.

The impact of storage conditions on RNA measurements and transcriptome profile is equally worth addressing. The integrity of RNA extracted from samples stored in RL had higher mean value compared to the RNA samples that were stored in LN_2._ This finding is in agreement with the previous finding by Bray et al.[14], where it was reported that RNA integrity is best maintained when tissues are stored in RL. Further analysis of the sequence data indicated that the ‘unique alignment’ percentage was significantly lower for samples that were stored in LN_2_. We also found evidences for differential gene expression in hypothalamus, liver and lungs, depending on the storage condition. Liver samples reacted the most to the storage condition as several genes appeared to be differentially expressed, upon storage in RL. The inability of RL to inhibit biological activity and thereby provide a stable gene expression [14, 15] might explain the differential gene expression in liver samples stored in RL.

Hypothalamus and lung samples that were stored in RL also showed differential gene expression, when compared to tissues stored in LN_2_. Several genes appeared to be differentially expressed in both these tissues that were stored in RL. We could not associate any meaningful interpretation while we searched further for gene ontology terms associated with these genes or while a pathway analysis was conducted on this limited number of differentially expressed genes. However, these observations clearly warrants that sample storage condition has the potential to cause a source of technical variation, especially while preserving samples for transcriptome studies. Perhaps, setting higher threshold to identify the differentially expressed genes will ensure that only the highly significant genes are detected in the analysis.

Our study was conducted in a small but accepted sample size for RNAseq studies, and it was specifically done in male piglets. This could be a source of bias and hence if future studies are conducted involving more samples representing both sexes, this can be ruled out. Further, quantseq method of analysis, where only the 3’end of the gene is sequenced, was used to detect the differentially expressed genes in our study. It would be interesting to see if our results are reproducible with other sequencing methodologies as well. However, we could already suggest that ANOXIA method of euthanasia might be a suitable alternative in research animals. We would also like to caution researchers about the impact of these often ‘forgotten’ variables, especially in gene expression studies. Storage medium like RNA*later* can influence the differential expression of genes.

## Conclusions

The ANOXIA method was found to be comparable to the T61 euthanasia method with regard to gene expression profiles in different tissues. This suggests that the nitrogen anoxia method could serve as a suitable alternative for euthanasia of piglets used in transcriptomic research. However, there could be some regulatory elements that can cause disruptions in the cellular processes and in turn alter the gene expression as was observed in the case of pituitary. Storage of samples in RL proved to better preserve RNA compared to storage in LN_2_, as indicated by higher RIN values. However, it’s worth noting that RL may be less effective in inhibiting biological activity. Consequently, the choice of storage conditions could potentially introduce confounding variables into our research findings. Researchers must be cautious of these potential sources of bias like euthanasia method and storage conditions, that could influence the research outcomes. Even if we are unable to prevent the effect of these external factors, it will be useful to identify the impact of these factors on the parameter under observation and thereby prevent misinterpretation of our research findings.

## Material and methods

### Sampling and RNA extraction

The experimental set up, method of euthanasia, sampling and storage conditions and RNA extraction protocol are described in Chakkingal Bhaskaran et al. [18]. In short, 12 one-week-old male piglets were used in the experiment, and 6 animals were euthanized at random either by ANOXIA or by T61. Samples were collected immediately, following euthanasia and confirmation of death. The study was conducted in male piglets since this trial also served as a pilot experiment on transcriptome analysis related to cryptorchidism. Tissue samples from pituitary, hypothalamus, lungs and liver were collected and later processed for transcriptome analysis. For pituitary, samples were only stored in RL, as the gland was too small to be split for the two conditions. Four samples from each tissue type per experimental condition (except lung sample (*n* = 3) from T61: LN_2_ group) were processed for the gene expression analysis. A detailed description of the samples (*n* = 59) used in the transcriptome analysis is given in the supplementary Table S5, Additional File 5.

### Library preparation and sequencing

Complementary DNA (cDNA) preparation, library preparation and sequencing was done at the Genomics Core facility of KU Leuven. Double stranded DNA (dsDNA) was constructed from the mRNA by random-primed reverse transcription and second-strand cDNA synthesis. Sequence libraries were prepared with the “3' mRNA-Seq library prep kit for Illumina (FWD)” from QuantSeq-Lexogen (Catn°015.96), following the manufacturer's protocol. Library quality and size range was assessed using a Bioanalyzer (Agilent Technologies, California, USA) with the DNA 1000 kit (Agilent Technologies, California, USA) according to the manufacturer's recommendations. Libraries were diluted to a final concentration of 2 nM and subsequently sequenced on a Illumina HiSeq4000 platform. Single-end reads of 50 bp length were generated with a minimum of around 2 million reads per sample. Quality control of raw reads was performed with FastQC v0.11.7 [38]. Adapters were filtered and trimmed off with ea-utils fastq-mcf v1.05 [39]. Seed based alignment was performed with HISAT2 version 2.1.0 [40] against the pig reference genome Sscrofa11.1, using the parameters (hisat2 -f -x genome -U reads.fa -S output.sam –no-spliced-alignment). Reads mapping to multiple loci in the reference genome were discarded. Resulting SAM alignment files were handled with Samtools v1.5 [41]. Quantification of reads per gene was performed with HT-seq Count v0.10.0 [42]. Analysis of Variance (One way ANOVA) and post hoc analysis (Tukey’s Honest Significant Difference (HSD) test) of the alignment data was used to assess the significance of difference between group means of the fixed factors. All sequences generated in this study is deposited to the Sequence Read Archive (SRA), with accession numbers SRR25184123 to SRR25184181 (BioProject number: PRJNA992446).

### Differential expression analysis

Count-based differential expression analysis was done with the Bioconductor package DESeq2 [43] in R. Samples representing different tissue types and conditions were normalized for differences in their sequencing depth. The size factor for each sample was calculated and the count data were divided by the size factor for normalization. The count data were further transformed using the function ‘*vst’* for variance stabilization, before visualization and clustering. Reported p-values were adjusted for multiple testing with the Benjamini–Hochberg procedure, which controls false discovery rate (FDR) [44]. The cut-off values for log2FoldChange (LFC), p-values and p-adjusted values for a sample size ranging between 4–12 is |0.5 – 2.0|, 10e-6 and 0.1 respectively, as recommended by Love et al. [43] and Schurch et al. [30]. The statistical model used in the analysis included the main effects of euthanasia method and storage condition, as well as the term indicating the interaction effect (euthanasia:storage). However, the model was re-run without the interaction term when it was found to be statistically non- significant.

### Supplementary Information

**Additional file 1:** **Fig. S1.** Comparison of mean ‘Unique Alignment Percentage’ between different tissue types. This figure illustrates the comparison of the mean ‘Unique Alignment Percentage’ across various tissue types - Pituitary, Hypothalamus, Lungs and Liver. The results of the post hoc analysis confirm significant differences between these tissue types, with the average ‘Unique Alignment Percentage’ in the hypothalamus being notably different from other tissue types. In the figure, Ranges (bars) and means (dots) indicate the spread and the average values of the ‘Unique Alignment Percentage’ respectively. Groups carrying different superscript letters (a, ab, b and c) and colours differ significantly (*p* < 0.05).

**Additional file 2:** **Fig. S2.** Comparison of mean ‘Unique Alignment Percentage’ between different storage conditions. This figure illustrates the comparison of the mean ‘Unique Alignment Percentage’ across different storage conditions - RNA*later *(RL) and LN2 (Liquid Nitrogen). The result of the post hoc analysis confirms significant differences between the two storage conditions, with the average ‘Unique Alignment Percentage’ for samples snap frozen in LN2 being significantly lower. In the figure, Ranges (bars) and means (dots) indicate the spread and the average values of the ‘Unique Alignment Percentage’ respectively. Groups carrying different superscript letters (a and b) and colours differ significantly (*p* < 0.05).

**Additional file 3:** **Table S1.** Results of analysis of variance (One Way ANOVA) for ‘Unique Alignment’ of reads to reference genome, according to different sources of variation.

**Additional file 4:** **Table S2.** Detailed summary of samples used in the study, with RNA quality parameters as well as complete information of sequence alignment.

**Additional file 5:** **Table S3.** List of differentially expressed genes ((LFC>= |0.5 - 2.0|, padj <= 0.1), detected on comparison of storage methods (RL vs LN2) in hypothalamus tissues.

**Additional file 6:** **Table S4.** List of differentially expressed genes ((LFC>= |0.5|, padj <= 0.1), detected on comparison of storage methods (RL vs LN2) in liver tissues.

**Additional file 7:** **Table S5.** Overview of samples selected for Quantseq Analysis - Samples were grouped by combining the factors euthanasia method and storage Condition.

## Data Availability

All transcriptome data generated in this study are deposited in the NCBI repository and are available in SRA under the bioproject PRJNA992446 (https://www.ncbi.nlm.nih.gov/bioproject/PRJNA992446).

## Abbreviations

RL: RNA*later*™
LN_2_: Liquid nitrogen
RIN: RNA integrity number
CO_2_: Carbon dioxide
SD: Standard deviation
IQR: Interquartile range
snRNAs: Small nuclear RNAs
LFC: Log2 fold change
snRNP: Small nuclear ribonucleoproteins
cDNA: Complementary DNA
mt*CO1*: Mitochondrial cytochrome C oxidase subunit 1
*CCK*: Cholecystokinin
*QSOX1*: Quiescin sulfhydryl oxidase 1
GO: Gene ontology
