# Supplementary material for: The forgotten variable? Does the euthanasia method and sample storage condition influence an organisms transcriptome – a gene expression analysis on multiple tissues in pigs
Source: BMC Genomics. 2023 Dec 14;24:769. doi: 10.1186/s12864-023-09794-4 (PMC10720124; doi:10.1186/s12864-023-09794-4)
Supplement: Supplementary file 1 — Additional file 1: Fig. S1. Comparison of mean ‘Unique Alignment Percentage’ between different tissue types. This figure illustrates the comparison of the mean ‘Unique Alignment Percentage’ across various tissue types - Pituitary, Hypothalamus, Lungs and Liver. The results of the post hoc analysis confirm significant differences between these tissue types, with the average ‘Unique Alignment Percentage’ in the hypothalamus being notably different from other tissue types. In the figure, Ranges (bars) and means (dots) indicate the spread and the average values of the ‘Unique Alignment Percentage’ respectively. Groups carrying different superscript letters (a, ab, b and c) and colours differ significantly (p < 0.05). [file 12864_2023_9794_MOESM1_ESM.docx]

***Table S1****: Results of analysis of variance (One Way ANOVA) for ‘Unique Alignment’ of reads to reference genome, according to different sources of variation*

| ***Source of Variation*** | ***df*** | ***Sum of Squares*** | ***Mean Square*** | ***F Value*** | ***Pr(>F)*** |
| --- | --- | --- | --- | --- | --- |
| Euthanasia Method | 1 | 3.31 | 3.305 | 0.4643 | 0.4985920 |
| Storage Condition | 1 | 92.38 | 92.379 | 12.9763 | 0.0006959 *** |
| Tissue type | 3 | 352.72 | 117.575 | 16.5155 | 1.058e-07 *** |
| Residuals | 53 | 377.31 | 7.119 |  |  |

Significance codes: 0 ‘***’ 0.001 ‘**’ 0.01 ‘*’ 0.05
