# Supplementary figures and images for: The forgotten variable? Does the euthanasia method and sample storage condition influence an organisms transcriptome – a gene expression analysis on multiple tissues in pigs
Source: BMC Genomics. 2023 Dec 14;24:769. doi: 10.1186/s12864-023-09794-4 (PMC10720124; doi:10.1186/s12864-023-09794-4)

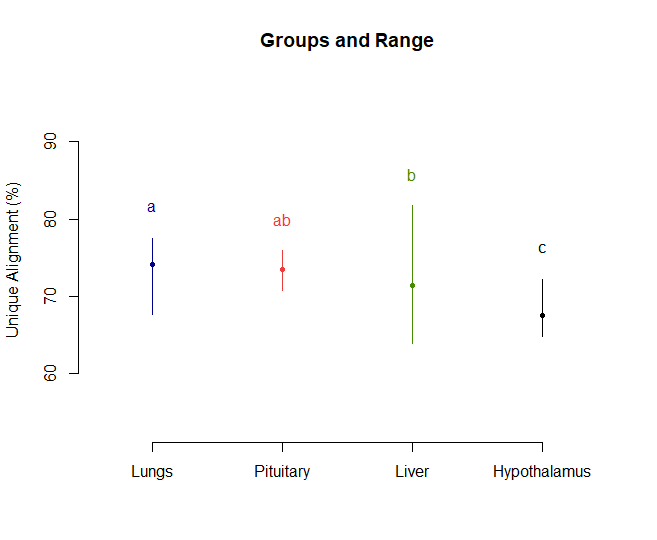

Supplement: Supplementary file 6 — Additional file 6: Table S4. List of differentially expressed genes ((LFC>= |0.5|, padj <= 0.1), detected on comparison of storage methods (RL vs LN2) in liver tissues. [file 12864_2023_9794_MOESM6_ESM.tiff]

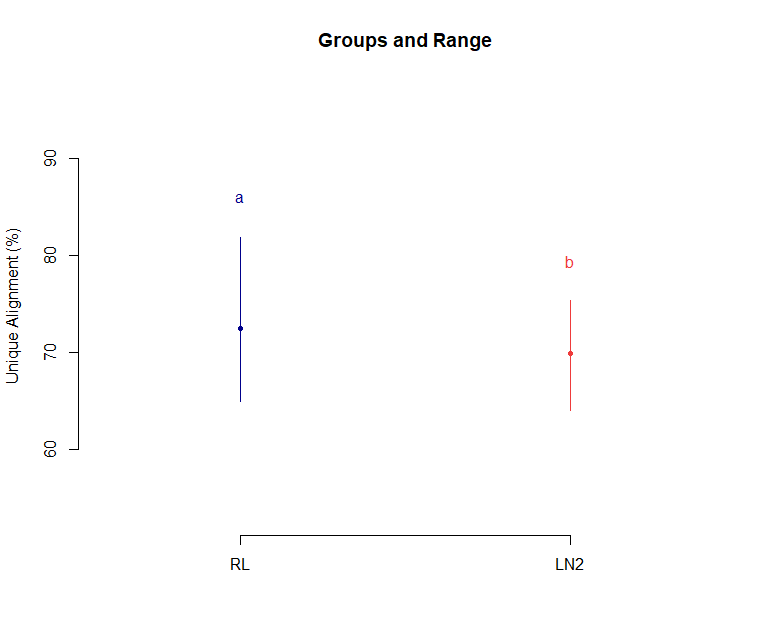

Supplement: Supplementary file 7 — Additional file 7: Table S5. Overview of samples selected for Quantseq Analysis - Samples were grouped by combining the factors euthanasia method and storage Condition. [file 12864_2023_9794_MOESM7_ESM.tiff]
